# Molecular Dynamics Simulations of the Host Defense Peptide Temporin L and Its Q3K Derivative: An Atomic Level View from Aggregation in Water to Bilayer Perturbation

**DOI:** 10.3390/molecules22071235

**Published:** 2017-07-22

**Authors:** Andrea Farrotti, Paolo Conflitti, Saurabh Srivastava, Jimut Kanti Ghosh, Antonio Palleschi, Lorenzo Stella, Gianfranco Bocchinfuso

**Affiliations:** 1 Dipartimento di Scienze e Tecnologie Chimiche, Università di Roma “Tor Vergata”, Rome 00133, Italy; farrox86@gmail.com (A.F.); paolo.conflitti@gmail.com (P.C.); antonio.palleschi@uniroma2.it (A.P.); stella@stc.uniroma2.it (L.S.); 2 Molecular and Structural Biology Division, CSIR-Central Drug Research Institute, Sector 10, Jankipuram Extension, Sitapur Road, Lucknow 226031, India; thesaurabhsrivastava@yahoo.co.in (S.S.); jk_ghosh@cdri.res.in (J.K.G.)

**Keywords:** LPS, Temporin, molecular dynamics simulations, Potential of Mean Force (PMF), peptide aggregation

## Abstract

Temporin L (TempL) is a 13 residue Host Defense Peptide (HDP) isolated from the skin of frogs. It has a strong affinity for lipopolysaccharides (LPS), which is related to its high activity against Gram-negative bacteria and also to its strong tendency to neutralize the pro-inflammatory response caused by LPS release from inactivated bacteria. A designed analog with the Q3K substitution shows an enhancement in both these activities. In the present paper, Molecular Dynamics (MD) simulations have been used to investigate the origin of these improved properties. To this end, we have studied the behavior of the peptides both in water solution and in the presence of LPS lipid-A bilayers, demonstrating that the main effect through which the Q3K substitution improves the peptide activities is the destabilization of peptide aggregates in water.

## 1. Introduction

Host Defense Peptides (HDP) are molecules present in many organisms, including humans [1]; they constitute the first protection against different pathogens, such as Gram-positive Gram-negative bacteria [2], viruses [3], fungi and yeasts [4,5]. More recently, an interesting anticancer activity of some HDPs has been observed [6,7,8,9]. The first peptide with antimicrobial activity has been discovered in 1939 [10,11]; since then, over 2600 different HDP sequences have been identified [12,13]. Several HDPs possess the ability to neutralize pro-inflammatory responses stimulated by bacterial debris, principally lipopolysaccharides (LPS) [14]. Moreover, HDPs can modulate immune responses, being responsible for different processes, such as chemokine production, and acting as promoters of adaptive immunity [4].

Many HDPs exert their anti-pathogen activity by permeabilizing the host-membranes [15]; as a consequence of their target, development of bacterial resistance is much less likely than for traditional antibiotics [16]. This evidence makes HDPs a promising class of antimicrobial drugs. However, many details of the processes leading to membrane permeabilization remain elusive [13,17] and different models have been proposed in order to describe their mode of action [18]. Among others, in the barrel-stave model, the active state is formed by aggregates in which the peptides assume a transmembrane orientation and assembly in a way that recalls the staves of a barrel [19,20]. An alternative view is provided by the carpet model [21,22,23] in which peptides bind to the membrane surface. After a threshold of membrane-bound concentration is reached, the perturbation induced in the outer leaflet of the membrane leads to the formation of water-filled defects and, eventually, membrane micellization. Many other models have been put forward, and, often, different mechanisms have been proposed for the same peptide [24]. This lack of clarity is mainly due to the difficulties involved in the application of the most common structural techniques (i.e., X-ray crystallography and NMR spectroscopy) to peptide/membrane systems [18,25]. Therefore, alternative approaches are particularly useful in this field, including Molecular Dynamics (MD) simulations, which can provide both atomic-level structural data and detailed pictures of the dynamic features of the peptide/membrane interaction [18,26,27,28,29,30,31,32,33]. In the last years, we have used MD simulations to study different HDPs, demonstrating the usefulness of simulative techniques, particularly if complemented with spectroscopic data. In these studies, we have clarified different aspects of the peptide/membrane interaction and their correlation with the peptide properties, both in the homogeneous phase [34,35,36,37,38,39,40] and in the bilayer [40,41,42,43,44]. In particular, we have investigated peptide/membrane interactions by using different simulative approaches, demonstrating the efficacy of Umbrella Sampling MD (US-MD) simulations to obtain reliable Potential of Mean Force (PMF) profiles for the peptide insertion into the bilayer [41]. Our works contributed to consolidate the idea that the activity of the HDPs on the bacterial membranes can be considered as the resultant of different factors. For instance, aggregation in water shields the hydrophobic moieties from the solvent, thus reducing the driving force for membrane binding [40]. In addition, once bound to the membrane different peptides perturb the bilayer to varying extents [43], and, in some cases, the effects on the membrane can be influenced by the experimental conditions (e.g., pH) [41,45]. 

**Scheme 1:**
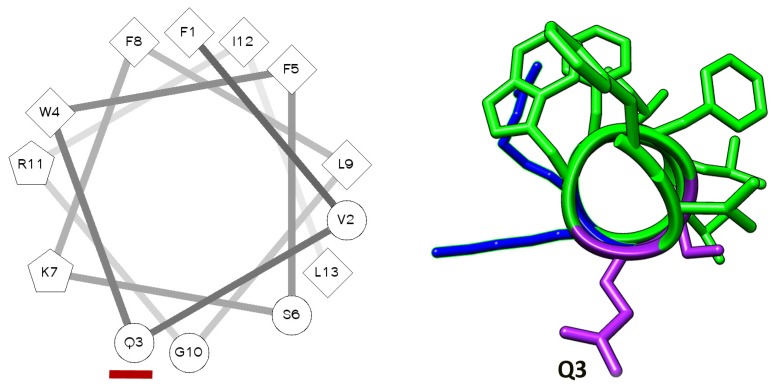
(**Left**) Helical wheel representation of TempL: In the scheme, the hydrophilic, hydrophobic and potentially positively charged residues are reported as circles, diamonds and pentagons, respectively. The third residue is marked with a red bar. (**Right**) View along the helix-axis of the TempL 3D helical structure: The side chains of the leucine, isoleucine, valine, phenylalanine and tryptophan residues are reported as green sticks; those of arginine and lysine are reported in blue; and those of glutamine and serine in violet.

We have also investigated short but very active peptides [34,35,36,38,42,43,44]. The shortness is an important feature to consider in view of possible therapeutic applications and temporins are very interesting under this respect. Temporins are a large family of HDPs isolated from the skin of frogs [46], comprises more than 70 short (10–14 residues) amphipathic peptides, with a net charge ranging from 0 to +4 at physiological pH [5]. Among the members of this class, Temporin-L (TempL), comprising 13 residues (Scheme 1), exhibits a high antimicrobial activity against both Gram-negative and Gram-positive bacteria, and yeast strains [47]. TempL aggregates in water [48,49] and it shows a strong affinity for lipopolysaccharides (LPS), being able to neutralize the pro-inflammatory responses caused by their release from the killed bacteria [50]. Unfortunately, TempL is also significantly hemolytic [49]. In vitro studies have shown a different mode of interaction of TempL with micelles mimicking bacterial and eukaryotic membranes [51]. In the former case, by using amphiphilic molecules with a net charge on the polar head, TempL localizes at the interface with the water solution. On the contrary, in the case of zwitterionic micelles, TempL is localized in the hydrophobic region of the bilayer. Based on these evidences, the authors suggest that TempL could follow a carpet or a barrel stave mechanism with prokaryotic and eukaryotic cells, respectively [49]. However, the outer membrane of the Gram-negative bacteria is constituted by lipopolysaccharides (LPS), and the orientation of TempL in LPS bilayers remains elusive.

In the attempt to augment the TempL antibacterial properties and its ability to neutralize pro-inflammatory responses, possibly also decreasing cytotoxicity, amino acid substitutions have been introduced in the wild type sequence [50,52,53]. One of us demonstrated that the Q3K substitution in TempL (Q3K-TempL) increases the ability to permeabilize LPS vesicles and augments the neutralization of LPS-induced pro-inflammatory response; at the same time, the Q3K substitution significantly reduces TempL aggregation in water [50]. Table 1 summarizes these results.

Little is known at the molecular level about the structural factors leading to these differences. In any case, peptide-LPS interaction possesses significant influence on antiendotoxin, LPS-permeabilizing and antimicrobial activities of a peptide [54,55]. This aspect has been specifically investigated in this study by using MD simulation. The obtained results are presented in three main sections, summarized below, which are related to three different aspects that can concur to the observed differences in antimicrobial and antiendotoxin activities between TempL and Q3K-TempL: (i) aggregation in water; (ii) interaction with LPS bilayers; and (iii) membrane-perturbing effects.

## 2. Results

### 2.1. Aggregation of the Peptides in Water Solution

The aggregation behavior in water of the peptides TempL and its variant Q3K-TempL has been evaluated by performing two independent simulations for each peptide. At the beginning of each MD simulation, eight peptides were randomly inserted in the simulation box, in a canonical α-helical conformation, obtaining two different starting points for each investigated system To test the reliability of our simulative approach, the same protocol was applied also to two recently reported TempL-derivatives, in which two phenylalanine residues at positions 5 and 8 were altered to leucine (F5,8L-TempL) and alanine (F5,8A-TempL) residues. Experimental data showed that the former has to some extent higher aggregation propensity than TempL, while the latter aggregates significantly less than the parent peptide [53].

**Figure 1:**
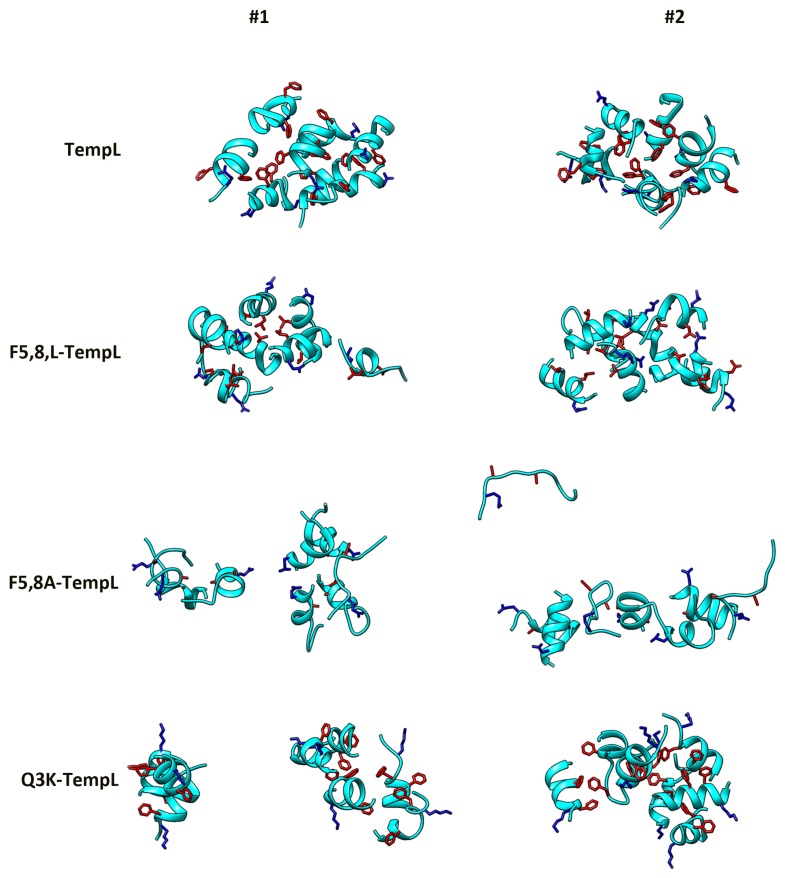
Final structures of the MD simulations in water. For each peptide (TempL, F5,8L-TempL, F5,8A-TempL and Q3K-TempL from the top) the structures from two independent simulations are reported. Peptides are represented as cyan ribbon; and residues 3, 5 and 8 are reported in stick representation (blue for Q/K residues at position 3 and red for the F/L/A at positions 5 and 8).

In all cases, during the MD trajectories, inter-peptide interactions took place very quickly and roughly 10 ns were needed to reach a stable configuration, which persisted essentially unchanged during the remaining simulation time. Figure 1 reports the structures obtained at the end of each simulation. In both TempL simulations, a single aggregate formed, comprising all eight peptides. The obtained aggregates were compact, with the phenylalanine residues interacting with each other and shielded from the solvent. A similar behavior was observed in the case of simulations with F5,8L-TempL. On the contrary, both the peptides F5,8A-TempL and Q3K-TempL showed a lower tendency to aggregate. In one of the two final structures of Q3K-TempL, the peptides did not form a single aggregate. A similar behavior was observed in both the simulations with F5,8A-TempL; furthermore, in the case of F5,8A-TempL, helical structures were less populated than for the other analogs.

To evaluate the features and the stability of the formed aggregates, the last 5 ns of the simulation trajectories have been analyzed (Table 2). The solvent accessible surface (SAS) of the aggregates is sensitive to both the compactness of the aggregates and to the number of separate aggregates formed [56]. Coherently with the inspection of the final structures reported in Figure 1, the SAS values for TempL and F5,8L-TempL are significantly lower than for F5,8A-TempL and Q3K-TempL. For Q3K-TempL, the higher value of the standard deviation reflects the different behavior observed in the two simulations, which is evident also in the two final structures reported in Figure 1. Of note, when singularly considered, both these simulations show an average SAS value greater than TempL and F5,8L-TempL (91 ± 2 and 81 ± 2 for simulations 1 and 2, respectively). 

A different way to check the presence and the compactness of aggregates in MD simulations is the measure of the average value of the distances between the centers of mass (COMs) of all the possible pairs of molecules [39]. For all the four investigated peptides, the starting values of this parameter were greater than 3 nm and then they decreased during the simulations; the values registered in the last 5 ns of the simulations are reported in Table 2. Similar to the SAS values, TempL and F5,8L-TempL show a lower value of the average distances between peptide pairs than F5,8A-TempL and Q3K-TempL, confirming the different stability of the obtained aggregates. To investigate these differences more in detail, we have searched for features that correlate with the different stability of the aggregates, by analyzing the effects of the amino acid substitutions on the peptide conformation and flexibility. Table 2 reports the percentage of the residues in the peptides that populate a helical conformation and the average RMSF values. 

In terms of RMSF, TempL and F5,8L-TempL show a lower mobility, probably a consequence of the high stability of their aggregates. A higher average RMSF value is observed for both F5,8A-TempL and Q3K-TempL, with the highest value observed for the former one. In terms of helicity, the F5,8L and Q3K substitutions have no effect. On the other hand, the F5,8A substitutions decrease the helical stability of the peptide.

Finally, Table 2 compares the SAS values of the modified residues (residue 3 for Q3K-TempL and residues 5 and 8 for both F5,8L-TempL and F5,8A-TempL). The greatest effect is observed in the case of the Q3K substitution, with a greater SAS registered for the lysine in Q3K-TempL with respect to glutamine in TempL.

Overall, under the simulated conditions the data show that F5,8L-TempL aggregates similarly to the parent peptide, TempL. In agreement with the available experimental data [53], the largest reduction in aggregate stability was observed for both Q3K and F5,8A substitutions but only for F5,8A-TempL was registered also a minor tendency to aggregate. By contrast, Q3K-TempL showed an augmented exposure of the mutated residues. Considering the obvious increase in electrostatic repulsions introduced by the latter mutation, the mechanism of aggregate destabilization might be different in the two cases.

### 2.2. Binding Energy of TempL and Q3K-TempL to Bilayers Mimicking the Outer Membrane of Gram-negative Bacteria

To investigate the binding free energy of both TempL and Q3K-TempL with LPS, the PMF profile of peptide insertion into a lipid-A bilayer has been evaluated. 

LPS is a very large and complex molecule, whose structure can be divided into an outer glycan polymer (*O*-polysaccharide), a core oligosaccharidic domain, and lipid-A, a phosphorylated glucosamine disaccharide decorated with multiple fatty acids, which is responsible for anchoring LPS to the outer membrane of bacteria. Here, we limited our simulative studies to a lipid-A bilayer. This choice strongly reduces the complexity of the system, and is justified by the experimental observation that TempL binding to LPS or to lipid-A is similar [57]. In addition, many of the immune activating abilities of LPS can be attributed to the lipid-A unit.

Insertion of a single peptide molecule has been studied, again for the sake of simplifying the simulated system. As shown above, temporins have a strong tendency to aggregate in water, but it has been reported that TempL tends to disaggregate in the presence of LPS [48]. NMR studies suggested a dimeric state for the peptide embedded in LPS micelles [49,58], but this could be induced by the high concentrations needed for NMR studies. Our data can be considered as representative of high dilution conditions.

**Figure 2:**
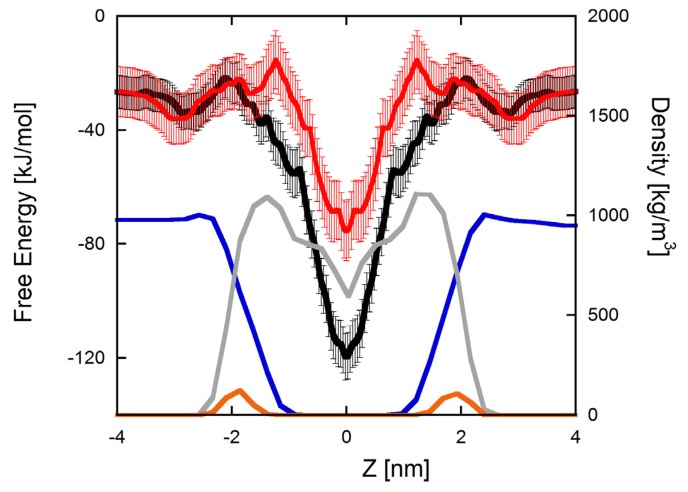
PMF profiles of the insertion of TempL (black) and Q3K-TempL (red) into a bilayer formed by Lipid-A. As reference, the density profiles of different parts of an unperturbed lipid-A bilayer are also reported. In particular, the water density profile is reported in blue and that of lipid tails and phosphate groups are reported in gray and orange, respectively. Roughly, the two peaks of the phosphates represent the boundaries of the membrane environment.

Figure 2 reports the PMF profiles obtained for the two peptides. In both cases, the global minimum is in the center of the bilayer, but differences are evident in the region comprised between 1.0 and 2.0 nm, where a maximum followed by a local minimum is present for Q3K-TempL, which is absent for TempL. This indicates that the Q3K change stabilizes a configuration with the peptide located just below the lipid-A saccharide moiety, which is absent in the parent peptide. However, these differences do not change significantly the configuration of the global minimum, notwithstanding the insertion of a positive net charge in the N-terminal region. 

A further difference between the two profiles is the depth of the global minimum, which is not as deep as for Q3K-TempL than for TempL, indicating that the interaction of the peptide with the bilayer is thermodynamically less favored for Q3K-TempL than for TempL. This is not surprising, considering that, in both cases, the peptide is embedded into the hydrophobic region of the bilayer and the amino acid change introduces an additional positive charge. The presence of the maximum discussed above and centered at roughly ±1.2 nm for Q3K-TempL suggests that its insertion could also be less favored from a kinetic point of view, with respect to TempL.

### 2.3. Effects of the TempL and Q3K-TempL Insertion on the lipid-A Bilayer

**Figure 3:**
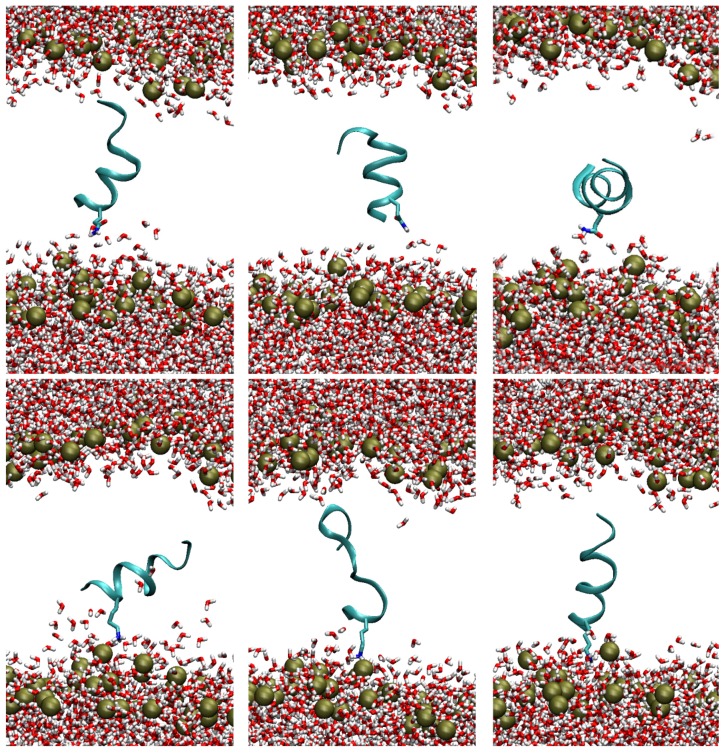
Representative structures for each one of the three clusters that characterize the configurations sampled in the global minimum of the PMF profile, as obtained using the g_cluster tool in the GROMACS software package. The structures reported above refer to the TempL peptide, those reported below to Q3K-TempL. The phosphorus atoms of the lipid-A, marking the position of the lipid polar heads, are indicated as gold spheres. The peptide backbone is reported as a cyan ribbon, the water molecules and the third residue of the peptides (Q and K in TempL and Q3K-TempL, respectively) are reported as sticks, with O, N and H atoms colored in red, blue and white, respectively. For the sake of clarity, the lipid tails are not reported.

To evaluate the effects on the membrane of the peptide insertion, the configurations populating the absolute minimum of the two PMF profiles have been analyzed. In particular, the structures in which the center of mass of the peptide lies within ±0.1 nm of the global minimum have been selected. This range, in both PMF profiles, corresponds to a ΔΔG of 1 kcal/mol with respect to the minimum. A clustering analysis of the ensembles created with this criterion identified three different equally populated families of configurations, for both TempL and Q3K-TempL. The most representative structures of each cluster are reported in Figure 3. Visual inspection of the three structures suggests that, despite of the localization of the peptides in the hydrophobic core of the bilayer, in both the analyzed peptides the third residue remains in contact with the water phase. This behavior is more pronounced in the case of the K residue in Q3K-TempL and seems to be coupled with both a deeper perturbation of the bilayer (in terms of water penetration and position of the lipid polar heads) and a lower stability of the peptide helical conformation, as confirmed by the amount of helical content in all the peptide conformations that fall in the PMF global minimum. These values resulted equal to 46% and 31% for TempL and Q3K-TempL, respectively, thus confirming that the insertion of a positive net charge in the side chain of residue 3 determines a lower stability of the helices in the membrane environment, contrary to the tendency registered in the water solution, in which differences in helical stability were not observed (Table 2).

**Figure 4:**
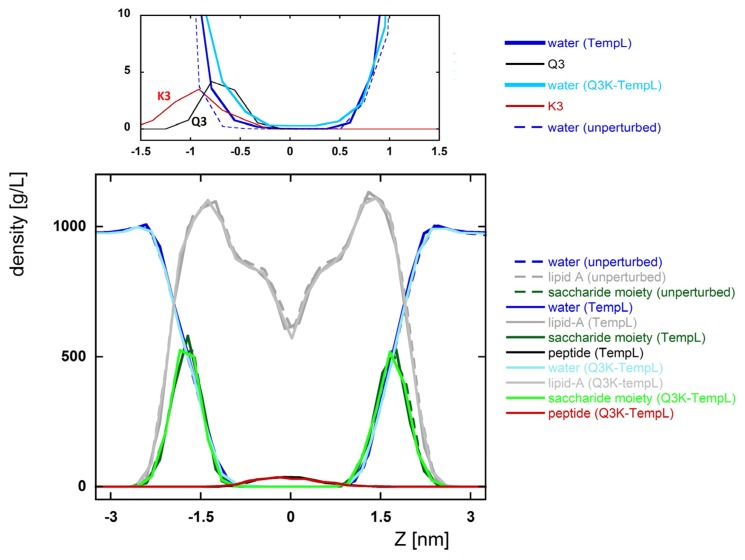
Density profile along the axis perpendicular to the bilayer plane (Z axis) calculated on the structures in the absolute minimum of the PMF profile. The reported profiles refer to water (broken blue, solid blue and solid cyan for unperturbed bilayer, with TempL and with Q3K-TempL, respectively), lipid-A (broken gray, solid gray and solid light gray for unperturbed bilayer, with TempL and with Q3K-TempL, respectively), the saccharide moiety of lipid-A (broken dark green, solid dark green and solid light green for unperturbed bilayer, with TempL and with Q3K-TempL, respectively). The black and red lines refer respectively to the whole TempL and Q3K-TempL peptides (below) and to the Q3 and K3 side chain residues (above). To highlight the effects of the mutated residue, the graph above reports a magnification of the central region of the density profile.

The density profiles of water and different groups of atoms of the lipid-A, calculated along the axis perpendicular to the bilayer plane, are reported in Figure 4. For comparison, the corresponding density profiles evaluated on an unperturbed bilayer are also reported. The profiles give information on the perturbative effects introduced by the peptide. In the figure, a magnification of the central regions is shown, evidencing that the average position of the third residue, Q or K, is different, with the former more embedded into the hydrophobic core of the bilayer. In both cases, the water penetrates more deeply than in the unperturbed bilayer. Interestingly, this effect is evident only in the interface close to the N-terminal regions of the peptides, whilst the other interface is unchanged in the three cases. In the case of Q3K-TempL, the water density never becomes zero, indicating that the presence of the peptide induces a slight translocation of water molecules between the two leaflets of the bilayer. A similar behavior is observed neither for TempL nor in the unperturbed bilayer. Overall, both peptides perturb the bilayer, and Q3K-TempL is the peptide that induces the largest effects.

**Figure 5:**
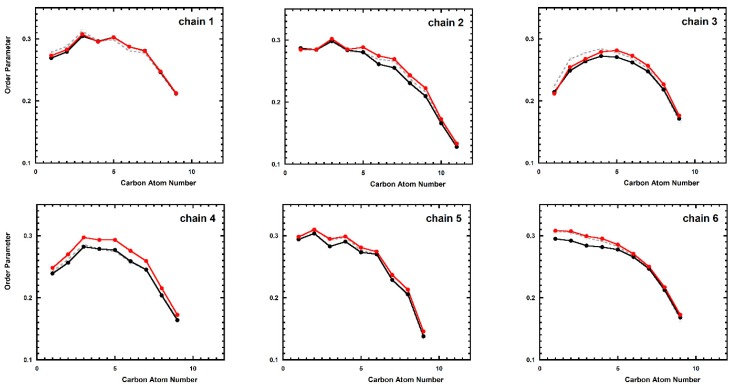
Deuterium order parameter for the six lipid chains of the lipid-A calculated starting from the structures in the global minimum of the PMF profile. Black, red and dashed-gray lines refer to TempL, Q3K-TempL, and unperturbed bilayer, respectively.

Finally, we have investigated the effects of the peptide on the lipid molecules in the bilayer. Figure 5 reports the order parameters of the lipid chains of lipid-A; for comparison, the data calculated on the unperturbed bilayer are also reported. The reported data do not show significant differences between TempL and Q3K-TempL. The results are essentially the same if the calculation is carried out separately on the lipids belonging to each of the two leaflets of the bilayer (data not shown).

## 3. Discussion

The activities of HDPs in terms of ability to permeabilize the membrane of pathogens and to reduce the toxicity of LPS, are dependent on different equilibria, which take place both in the water and in the lipid phase [40,42,59,60,61,62,63]. In this study, we have investigated different aspects related with antibacterial and antiendotoxin activities of the peptide TempL and its derivative Q3K-TempL. To this end, we have simulated separately aggregation of the peptides in water solution and their interaction with a lipid-A bilayer mimicking the Gram-negative outer membrane and the LPS-containing debris caused by bacterial killing and leading to endotoxin activity.

MD techniques were used in the past to investigate the TempL folding, without considering the aggregation properties investigated in the present work [64]. The tendency to aggregate in water causes a significant reduction in the antimicrobial activity of many HDPs. It has been shown that a preliminary crosslinking of active HDPs strongly impairs their ability to pass through the LPS membrane [65,66]. This is mainly a consequence of the reduced diffusion of the aggregates through the cell wall, due to their larger size in comparison to the corresponding monomers. In addition, aggregation reduces the effective peptide hydrophobicity, thus affecting the driving force for membrane binding. Of note, the aggregation state of these peptides is also related to their antiendotoxin activity [50,53]. In the present work, the propensity to aggregate in water of TempL and three different derivatives has been investigated by means of MD simulations. Our data are in good agreement with the experimental evidences, showing that the aggregation propensity of the investigated peptides follows the order F5,8L-TempL ≈ TempL > Q3K-TempL ≥ F5,8A-TempL [50,53], supportingthe reliability of our approach.

Beyond the differences in the aggregation state in water solution, our data shed light on the orientation of TempL into lipid bilayers (i.e., if it lies parallel to the water/lipid interface or if it is embedded into the hydrophobic region), which is still debated as different results have been reported, depending on the experimental conditions. The peptide orientation in the bilayer is crucial to understand the mechanism of pore formation. TempL behavior is particularly important because, among the temporins, it is the most active against Gram-negative bacteria [49]. Unfortunately, TempL shows also a marked activity against erythrocytes, which hampers its use as an antimicrobial drug. For this reason, many studies have been performed to understand and to modulate its interaction with different membranes. TempL has net charge (+3) that in principle makes possible a carpet mechanism, in which the peptides assume an interfacial position; on the other hand, the hydrophobic residues present in the TempL sequence could favor also a barrel-stave mechanism, with the peptides embedded into the hydrophobic core. The experimental evidences show that both superficial and embedded configurations are possible. In SDS micelles, an interfacial arrangement is preferred, whilst insertion in the hydrophobic core is registered if DPC lipids are used [51]. These evidences open the door to the possibility that TempL could follow different mechanisms when it permeabilizes neutral membranes of eukaryotes or the charged membranes typical of bacteria. NMR studies have shown that the N-terminal region of TempL is strongly involved in the insertion into neutral membranes [51] and attempts to impair this insertion have been done also by adding a net charge in this region with both Q3R [52] and Q3K [53] substitutions. Though Q3K-TempL retains the cytotoxic properties of TempL, it shows higher activity against Gram-negative bacteria and significantly improved anti-endotoxin property than the parent peptide (see Table 1). We have evaluated the effects of the Q3K substitution by calculating the PMF profile of the interaction of TempL or Q3K-TempL with a lipid-A bilayer, a simulative approach that we have successfully used to predict the effect of net charges on the peptide insertion [41]

The charge density of a lipid-A membrane (−2 for each molecule comprising six acyl chains) can be considered as intermediate between the zwitterionic polar head present on DPC micelles, which favor peptides embedded in the hydrophobic region, and the charge present on SDS molecules (−1 for each acyl chains), which stabilizes interfacial configurations (see above). For this reason, it is interesting to observe that in the most stable configuration of the PMF profiles both TempL and Q3K-TempL are deeply embedded into the bilayer. Under these conditions, the insertion of an additional net charge in the N-terminal region of TempL it is not enough to promote an interfacial localization of the peptide. It can be speculated that the same occurs with neutral bilayers, where the electrostatic terms at the interface are less important than for LPS, thus nullifying the attempt to make Q3K-TempL ineffective against eukaryotic cells. In addition, our data support the idea that also short peptides can assume stable monomeric state embedded into the hydrophobic region of bilayers, in spite of their length [42]. According to the PMF profiles, both TempL and Q3K-TempL can bind and translocate the Gram-negative outer membrane in the monomeric state. This is possible due to an intrinsic flexibility of the bilayer, which allows slight perturbations of the interface to “accommodate” the peptide in the lipidic phase. On the other hand, differences between TempL and Q3K-TempL are also observed. The depth of the global minimum in the PMF profile indicates for Q3K-TempL a minor tendency to bind to the membrane with respect to the parent peptide TempL, a result a priori difficult to predict, as usually the addition of a net charge in HDPs improves the binding to negatively charged bilayers. Overall, our data suggest that the higher tendency to depolarize LPS vesicles exhibited by Q3K-TempL with respect to TempL (Table 1) is not related with a higher affinity of the peptide for the membrane but is mainly due to the effects on the aggregation in water, which favor the monomeric state that in turn binds the membranes. Finally, in spite of the similar PMF profile, TempL and Q3K-TempL behave slightly differently when embedded in the membrane. Due to the presence of a net charge, the K3 side chain populates more superficial positions than Q3. This different behavior of the third residue produces a different stability of helical conformation in the two peptides, in particular, Q3K-TempL in the lipid-A bilayer populates more extended conformers and helices are less abundant than for TempL. Furthermore, the Q3K substitution induces greater perturbation on the bilayer with respect to TempL, as only in the simulations with Q3K-TempL water molecules has been detected in the hydrophobic core of the bilayer. Overall, these evidences suggest that both peptides can translocate and/or permeabilize the outer membranes of Gram-negative peptides, but the perturbation induced by Q3K-TempL is more evident than for TempL. Finally, the PMF profile of Q3K shows a local minimum followed by a maximum close to the interface, which is absent in the profile obtained with TempL. This minimum is not favored with respect to that at the center of the hydrophobic region; however, it cannot be ruled out that different conditions favoring peptide binding at the interface could change the relative weight of the two alternative orientations, thus determining more significant differences between TempL and Q3K-TempL.

In conclusion, in this work we have shown, once again, the importance of all the possible equilibria on the antimicrobial activity of the HDPs. In this context, MD simulations can give a structural and dynamical insight that is useful to rationalize the experimental evidences. In particular, in the case of TempL here investigated, our simulations suggest a counterintuitive effect of the substitution of the glutamine residue at the third position with a lysine. The higher activity of Q3K-TempL with respect to TempL for the negatively charged LPS bilayer is not due to an increase in the peptide/membrane affinity, as would be hypothesized by considering the addition of a positive charge on the peptide. The introduction of the charged residue at the N-terminal region of the peptide does not change the interaction mode with the membrane, and the peptide remains deeply embedded in the hydrophobic region of the bilayer; in this environment, the insertion of a net charge decreases the interaction energy. On the contrary, the higher tendency to depolarize LPS vesicles observed for Q3K-TempL, and the enhanced antimicrobial and antiendotoxin activities, are likely due to the effect of the substitution on aggregation in the water phase. We have shown that the Q3K substitution destabilizes aggregates in water, thus increasing the effective concentration of the monomer, which has a higher driving force for membrane binding due to the exposed hydrophobic residues.

## 4. Methods

### 4.1. Simulations in Water

Simulations were performed by using the GROMACS suite [67], with the ffG53a6 force field [68]. The MD settings were adopted according to previously described procedures [39], with slight changes. Briefly, the SPC model was used for water [69]. Following initial energy minimization and a 100 ps MD run during which the peptide atoms were position restrained, the temperature was raised to the value set in the simulation (300 K) in a stepwise manner, performing four MD runs, 50 ps each, at different temperatures (50, 100, 200, and 250 K). A Berendsen thermostat, with a coupling constant of 0.1 ps, was applied to keep temperature constant; the pressure was kept constant (1 bar) using a Berendsen isotropic barostat (1 ps time constant) [70]. Bond lengths were constrained with the LINCS algorithm [71]. Electrostatic interactions were calculated by using the particle mesh Ewald (PME) procedure [72], with a cutoff of 1.2 nm. Van der Waals interactions were calculated using a cutoff radius (1.2 nm). A time-step of 2 fs was employed. In the starting configuration an α-helical conformation was assumed for the eight peptides, which were randomly placed in a cubic box with side dimension of 6.3 nm containing roughly 3800 water molecules. Chloride anions were randomly added to neutralize the solutions. For each system, the simulations were 17 ns long, and replicated twice. The analyses were conducted on the last 5 ns of the simulations. The secondary structures were assigned by means of Dictionary of Protein Secondary Structures (DSSP) [73]. The helical content was calculated by considering the α, 3_10_ and π helices. To evaluate the Solvent Accessible Surface Area (SAS), the g_sas tool in GROMACS was used with the default settings. For the SAS of the residues, a percentage is reported, which is evaluated with respect to the values observed for the corresponding amino acids when considered as detached in water. The distances between the centers of mass (COM) of the peptides were evaluated by using the g_dist tool in GROMACS. At each time, the distances between all the 28 couples of peptides were calculated and averaged; these values were then averaged on the last 5 ns of the two simulations for each system. The Root Mean Square Fluctuations (RMSF) for each residue were calculated in the last 5 ns of simulations by using g_rmsf tool in GROMACS; the average of these values for each peptide has been reported. The figure reporting the final structures of each simulation was produced by using UCSF Chimera [74].

### 4.2. PMF Calculations

The interaction of TempL and Q3K-TempL with a lipid-A bilayer was investigated by determining the PMF profile of the peptide insertion after US-MDs [75]. Eighty-one independent simulations were carried out to obtain each PMF profile, in which the peptide center of mass (COM) was restrained at a specified distance from the bilayer COM (every 0.1 nm from +4.0 nm to −4.0 nm along the axis normal to the bilayer). A force constant of 500 kJ mol^−1^ nm^−2^ was imposed on the peptide COM. The simulations box contained the peptide, 72 lipid-A and roughly 15000 water molecules, Cl^−^ anions to neutralize the peptide charge (two for TempL, three for Q3K-TempL) and 72 Ca^2+^ cations to neutralize the lipid-A. The starting configurations, with the peptide embedded into the bilayer, were obtained according to a protocol developed in our laboratory [41,42]. The peptide starting structure was a perfect α-helical conformation, with the helix parallel to the membrane normal. The boxes were first equilibrated by using an annealing approach, with the temperature gradually increased from 50 K to 300 K, and then simulated for 24 ns at 300 K. PMF was calculated in the last 15 ns of simulation for each window. The Weighted Histogram Analysis Method (WHAM) [76] was used, using 50 bins and a tolerance of 10^−6^ kJ mol^−1^. Obtained profiles were finally symmetrized with respect to the bilayer center by using the g_wham GROMACS tool. The parameters for lipid-A were taken from Piggot and coworkers [77]. The same conditions used for the simulations in water were applied, with the exception of the cut-off in the PME and van der Waals calculations, which in this case were both equal to 1.4 nm. The deuterium order parameters, for each one of the six aliphatic chains of the lipid-A, were calculated by using the g_order tool in GROMACS. The density profiles along the bilayer normal were determined by means of the g_density tool in GROMACS. The center of the bilayer was calculated by symmetrizing the density profile of the aliphatic chains of lipid-A. The structural figure was produced by using VMD [78].

## Figures and Tables

**Table 1:** Sequences and properties ^1^ of TempL and of the Q3K analog.

Peptide (Sequence)	Net Charge (*e*)	Hydrophobicity (min) ^2^	Aggregation in Water ^3^	Antibacterial Activity (μM) ^4^	LPS Permeabilization (%) ^5^	Anti Endotoxin Activity (mg/kg) ^6^
TempL (FVQWFSKFLGRIL-NH_2_)	+3	21	17	10	24	1.0
Q3K-TempL (FVKWFSKFLGRIL-NH_2_)	+4	19	6.5	5	100	0.25

^1^ Data extracted from Srivastava and Ghosh [50]; ^2^ RP-HPLC retention time; ^3^ Fold increment in rhodamine fluorescence after Proteinase K treatment of labeled peptides. Aggregation quenches the fluorescence and peptide digestion removes this effect; ^4^ MIC against *E. coli*; ^5^ Percentage of recovery of the fluorescence intensity of the potential sensitive dye di-S-C_3-5_ after addition of 10 μM peptide to LPS vesicles with a transmembrane potential; ^6^ Minimum dose required to attenuate lethal endotoxemia in LPS treated balb/c mice.

**Table 2:** MD simulations in water. The reported parameters have been evaluated in the last 5 ns of the simulations, as average of two independent simulations.

Peptide	Solvent Accessible Surface (SAS)			Average COM Distance (nm)	RMSF (nm)	Helicity (%)
	Peptide ^1^ (nm^2^)	Residue 3 ^2^ (%)	Residue 5,8 ^2^ (%)			
TempL	79 ± 3	65 ± 4	24 ± 7	1.89 ± 0.04	0.26 ± 0.03	50 ± 7
F5,8L-TempL	79 ± 4	---	25 ± 6	1.9 ± 0.1	0.22 ± 0.06	50 ± 7
F5,8A-TempL	88 ± 2	---	29 ± 9	2.5 ± 0.1	0.35 ± 0.07	38 ± 4
Q3K-TempL	86 ± 6	86 ± 6	---	2.4 ± 0.4	0.30 ± 0.09	51 ± 6

^1^ The SAS is reported as absolute value; ^2^ The SAS is calculated as average on the two simulations and it is reported as percentage with respect to the SAS value of the amino acids in water.
